# The Effect of Expected Value on Attraction Effect Preference Reversals

**DOI:** 10.1002/bdm.2001

**Published:** 2016-12-19

**Authors:** George D. Farmer, Paul A. Warren, Wael El‐Deredy, Andrew Howes

**Affiliations:** ^1^ Division of Neuroscience and Experimental Psychology University of Manchester Manchester UK; ^2^ School of Biomedical Engineering University of Valparaíso Valparaíso Chile; ^3^ School of Computer Science University of Birmingham Birmingham UK

**Keywords:** attraction effect, contextual preference reversals, expected value, value‐maximising

## Abstract

The attraction effect shows that adding a third alternative to a choice set can alter preference between the original two options. For over 30 years, this simple demonstration of context dependence has been taken as strong evidence against a class of parsimonious value‐maximising models that evaluate alternatives independently from one another. Significantly, however, in previous demonstrations of the attraction effect alternatives are approximately equally valuable, so there was little consequence to the decision maker irrespective of which alternative was selected. Here we vary the difference in expected value between alternatives and provide the first demonstration that, although extinguished with large differences, this theoretically important effect persists when choice between alternatives has a consequence. We use this result to clarify the implications of the attraction effect, arguing that although it robustly violates the assumptions of value‐maximising models, it does not eliminate the possibility that human decision making is optimal. © 2016 The Authors Journal of Behavioral Decision Making Published by John Wiley & Sons Ltd.

## Introduction

The attraction effect (Huber, Payne, & Puto, 1982) refers to the puzzling change in preference that occurs when an apparently irrelevant alternative is added to a choice set. This decoy option, despite not being chosen, nonetheless changes preferences between existing members of the set. Over 30 years of research into the effect have shown it to be a robust phenomenon highlighting the impact of context on decision making. It has been found in choices among prospects (Herne, 1999; Soltani, De Martino, & Camerer, 2012; Wedell, 1991), low‐level perceptual decisions (Choplin & Hummel, 2005; Trueblood, Brown, Heathcote, & Busemeyer, 2013), motor planning decisions (Farmer, El‐Deredy, Howes, & Warren, 2015), inference tasks (Trueblood, 2012), choices between consumer products (Huber et al., 1982; Noguchi & Stewart, 2014; Simonson & Tversky, 1992), animal choice (Shafir, Waite, & Smith, 2002), and even decisions made by slime mould (Latty & Beekman, 2011). Despite the presence of the effect across species and paradigms, recent work has cast doubt on the robustness of the attraction effect in human consumer decision making (Frederick, Lee, & Baskin, 2014; Yang & Lynn, 2014). Frederick et al. (2014) argue that the effect may only be elicited when choice problems are described in terms of a numeric representation of their attribute values. Alternative representations such as images of products or pie charts representing gambles are shown not to elicit the effect.

**Figure 1:**
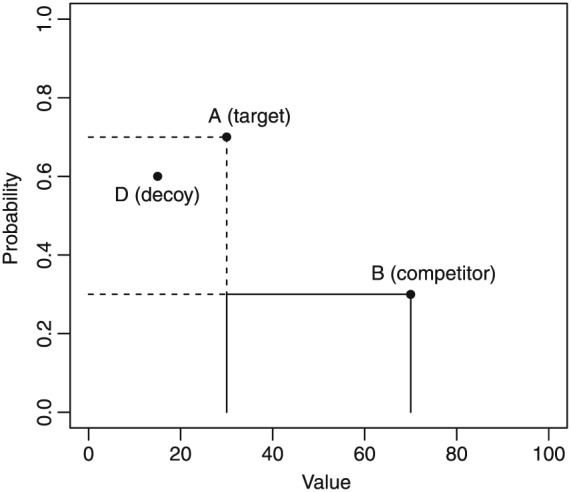
The attraction effect in choices among prospects. The decoy is dominated by the target on both attributes, but by the competitor on only one. In this figure, A is the target and B is the competitor. If the decoy were in the solid area rather than the dashed area, then B would be the target and A the competitor

In the standard form of the attraction effect, options in a choice set are described by two attributes that trade‐off, such as the probability and value of prospects, or the fuel‐economy and acceleration of a car. Two of the options are difficult to choose between because each dominates the other on one of the attributes. When a third decoy option is introduced, it is dominated by one of the original options (the target) on both attributes and by the other option (the competitor) on only one attribute (Figure 1).

Adding this asymmetrically dominated decoy has the effect of biasing choice away from the competitor toward the target. In a two‐option choice, each option might be chosen 50% of the time, but when the decoy is added, the target might be chosen 60% of the time, and the competitor 40% of the time. Note that the decoy is not chosen as it is obviously worse than the target. Despite this, its presence influences the ratio of choices between target and competitor.

Perhaps the biggest influence of the effect on the decision‐making literature has been the observation that it violates the axioms of regularity and independence from irrelevant alternatives, necessary in Luce's (1959) choice axiom and other value‐maximising models (Ariely & Wallsten, 1995; Heath & Chatterjee, 1991; Huber et al., 1982; Louie, Khaw, & Glimcher, 2013; Ratneshwar, Shocker, & Stewart, 1987; Roe, Busemeyer, & Townsend, 2001; Sen, 1998; Simonson, 1989; Tsetsos, Usher, & Chater, 2010; Tversky & Simonson, 1993; Usher & McClelland, 2004). This failure of rational models to account for the attraction effect has led to the interpretation that it is sub‐optimal: ‘people err by complicating rather than simplifying the task’ (Tversky & Simonson, 1993, p. 1188); ‘A limitation of rationality in choice preference’ (Usher, Elhalal, & McClelland, 2008, p. 297).

A number of theories of the choice process suggest that sub‐optimal behaviors arise because, rather than integrating attribute values in an expected value like calculation, people make ordinal comparisons between options. Only recently have these proposed processes been examined with eye‐tracking data (Noguchi & Stewart, 2014). These data suggest that people compare pairs of alternatives within the attended attribute, supporting accounts of context effects such as decision by sampling (Stewart, 2009) and the multi‐attribute linear ballistic accumulator model (Trueblood, Brown, & Heathcote, 2014). For example, in decision by sampling, the attraction effect is theorised to result from the comparison of pairs of alternatives within an attribute; as the target‐decoy comparison accumulates wins for the target on both attributes, whereas the competitor decoy comparison accumulates a win and a loss for the competitor, it is the target that benefits the most from the presence of the decoy.

Existing demonstrations of the attraction effect use decisions between alternatives that are approximately equally valuable. Indeed, many designs explicitly use target and competitor prospects that have the same expected value (Herne, 1999; Huber et al., 1982; Wedell, 1991), or that have subjectively equivalent expected values (Soltani et al., 2012). In perceptual decisions, Trueblood et al. (2013) found the attraction effect when participants were asked to select the rectangle with the largest area. The target and competitor stimuli presented, in fact, had the same area. This feature of these designs is intended to provide the most favourable environment for eliciting the attraction effect. Having the same expected value makes the options hard to choose between, so preferences may be less certain and more easily biased by the decoy. Indeed, there is some evidence for this as Mishra, Umesh, and Stem (1993) show that initial preference levels can predict the extent of the attraction effect.

In this paper, we argue that existing demonstrations of the attraction effect provide evidence that people violate the axioms of value‐maximising models, but *not* that they are sub‐optimal. Because these experiments typically use alternatives that are of approximately equal expected value to elicit the effect, participants will maximise utility in these experiments regardless of whether they choose the target or competitor. As long as they avoid a dominated decoy, all of their choices are, by virtue of the experimental design, utility‐maximising. The attraction effect, therefore, reveals a decision process that violates axioms required of value maximisation but fails to provide evidence that this occurs in situations where there is a material consequence. In the studies reported subsequently, we have addressed this issue by parametrically manipulating the expected value difference (EVD) between the two non‐decoy alternatives. The aim of the experiments is to demonstrate, for the first time, that preference reversals can cause participants to make non‐utility‐maximising choices. 1We manipulate the expected value difference between lotteries as a proxy for expected utility.


Many rational theories of choice are ‘as if’ models, silent on the mechanism that reaches the decision, concerned instead with the consequences of the decision. If the attraction effect can be elicited in the face of EVDs, then we can start to ascertain how important it is in terms of outcome, rather than what it tells us about choice mechanisms. This point is of critical importance to economists and proponents of rational value‐maximising accounts.

[we should] conduct experiments which show not merely that individuals are sometimes systematically non‐rational, but which would start to give us some feeling for how important this phenomenon might be. For example, I don't think that any of the preference reversal experiments have yet given information that would allow us to graph the frequency of preference reversals as a function of the difference in expected value (in, say, percentage terms) between the two gambles with which subjects are presented. While this information might not dramatically affect how investigators with very different theoretical dispositions evaluated the data, it would help address the issue of whether we are seeing a phenomenon likely to play an important role in natural environments (Roth, 1996, p. 202).

In what follows, we provide a rigorous test of the importance and robustness of the attraction effect using both explicitly described, and more perceptual representations of gambles. We discuss the implications of these results for theories of human choice. In particular, we argue that while contextual preference reversals appear *locally* irrational in the sense of violating axioms of decision models, they may in fact be *globally* rational when the decision environment and cognitive constraints are taken into account (Howes, Warren, Farmer, El‐Deredy, & Lewis, 2016).

### Overview of experiments

In Experiment 1, we devised a perceptual representation of lotteries and manipulated the absolute difference in expected value between target and competitor prospects. Participants made time‐limited choices and could only view one prospect at a time. In Experiment 2, we sought to replicate and generalise Experiment 1 to demonstrate that the effects observed were not task dependent. We used a relative (percentage) difference in expected value between the prospects and removed the time and serial viewing constraints. We further extended the design to include additional stimuli types including explicitly described gambles and an area judgment task based on Trueblood et al. (2013).

## Experiment 1

### Methods

#### Participants

Forty‐seven students and staff (14 male) from the University of Manchester volunteered to take part. Participants were between 21 and 53 years old (*M* = 27). Informed consent was collected, and participants were paid £7.00. The experiment took approximately 45 minutes to complete.

#### Design

We used a within‐subjects design to test the effect of EVD on the dependent variable of preference reversal rate. For each participant, preference reversal was measured as the proportion of target choices minus the proportion of competitor choices.

The EVD independent variable had four levels (0, 3, 6, and 9). In order to achieve four levels of EVD, eight prospects were chosen. Four (called the V prospects) were given a fixed probability of .2 and varying values to achieve expected values 8, 11, 14, and 17. The other four prospects (the P prospects) were given a fixed value of 25 and varying probabilities to achieve the same expected values as the V prospects (Table 1). Each V prospect had a higher value, but lower probability than each P prospect. Each of the V prospects was paired with each of the P prospects to create 16 choice sets. Prospect pairs with the same expected value had an EVD of zero, while, for instance, a prospect with expected value eight paired with a prospect with expected value 17 had an EVD of nine.

**Table 1:** Expected value difference between prospect pairs in Experiment 1

High probability prospects (P)	Low probability prospects (V)			
	.2 (40)	.2 (55)	.2 (70)	.2 (85)
.68 (25)	9	6	3	0
.56 (25)	6	3	0	3
.44 (25)	3	0	3	6
.32 (25)	0	3	6	9

Each of the choice sets was presented once with prospect P as the target and once with prospect V as the target resulting in 32 unique choice sets. Each of these unique choice sets was repeated eight times, creating a total of 256 trials per participant. The experiment was divided into four blocks of 64 trials. The presentation of the trials was completely randomised with the exception that two trials could not be consecutive if the only difference between them was the position of the decoy. This control was added to ensure that the experimental manipulation was not too obvious to the participants. The decoy position defined whether prospect V or prospect P was the target. The decoys were always of the same value as their target but with 10% lower probability.

#### Stimuli

**Figure 2:**
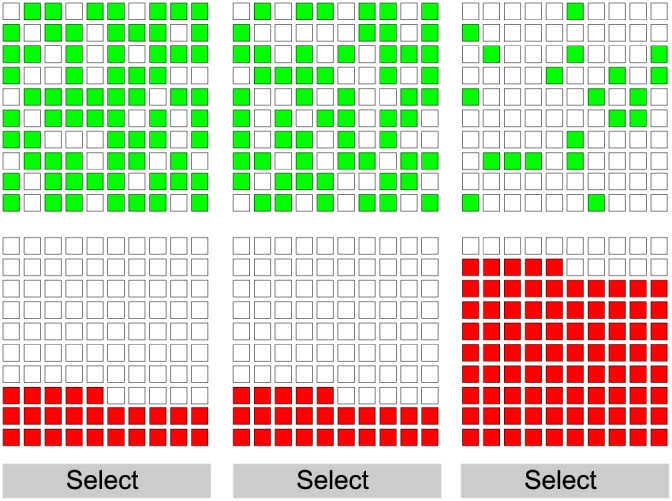
The stimuli used in Experiment 1. The density of green squares (top row) represents the probability of the prospect. The number of red squares (bottom row) represents the value of the prospect. Each column represents an alternative prospect. The probability and value of only a single prospect are displayed at any one time. The participant chooses a prospect by pressing the corresponding ‘select’ button. [Colour figure can be viewed at wileyonlinelibrary.com]

Participants were asked to choose between three prospects. Each prospect had a probability *p* of winning a value *v* in the form *p*(*v*) or (1 − *p*)(0). The probability of each prospect was presented to participants using a grid consisting of 100 squares (Figure 2). For a prospect with a success probability of .6, 60 of the squares were shaded green. For a success probability of .4, 40 of the squares were shaded green, and so on. The position of the green squares within the grid was randomised. The value of each prospect was presented in an identical 10 by 10 grid immediately below the probability information. For the value grid, the display was not randomised and was shaded red. Forty red squares indicated a value of 40, 70 red squares a value of 70, and so on. There were six grids in total, one probability and one value grid for each of the three prospects.

#### Procedure

At the start of each trial, all of the displays were blank. Participants revealed the probability and value information for each prospect by holding the mouse button down in a blue shaded area between the probability and value displays. Lifting off the mouse button cleared the display. Participants could only view one prospect at a time but could choose any order to view them. This prevented participants from assessing alternative prospects by making a perceptual comparison of the visual density of the different displays, encouraging them instead to encode the information. In each trial, participants were given 5 seconds of viewing time that they could distribute between each of the prospects as they saw fit. A 5‐second countdown timer was displayed on the left of the interface. The timer only counted down while the participants had the mouse button depressed, and the probability and value information were visible. Participants could only make their choice once the timer had reached zero. An enforced assessment duration allowed us to control the amount of effort participants put into each trial. Participants were asked to choose the prospect they preferred by clicking a button marked ‘Select’ that was positioned below the value grid for each of the prospects. Participants did not receive any feedback on their decisions.

### Analysis

As noted, in order to determine the preference reversal rate, we subtracted the proportion of competitor choices from the proportion of target choices. This metric was averaged across the prospect pairs for each level of EVD. A positive difference indicated that participants preferred a prospect more often when it was the target than when it was the competitor; this is the outcome that is expected when measuring the attraction effect. A negative difference indicated that participants preferred a prospect more often when it was the competitor than when it was the target. No difference indicated that participants were consistent and chose a prospect the same number of times regardless of whether it was the target or competitor.

### Results

To check that participants perceived the decoy option as inferior to the target option, we examined decoy selections for each of the 16 choice sets in both contexts. Participants sometimes chose the decoy option instead of the target or competitor. Five participants were excluded from all analyses as their decoy selection rate exceeded 2.5 times the median absolute deviation (Leys, Ley, Klein, Bernard, & Licata, 2013).

#### Effect of expected value difference

**Figure 3:**
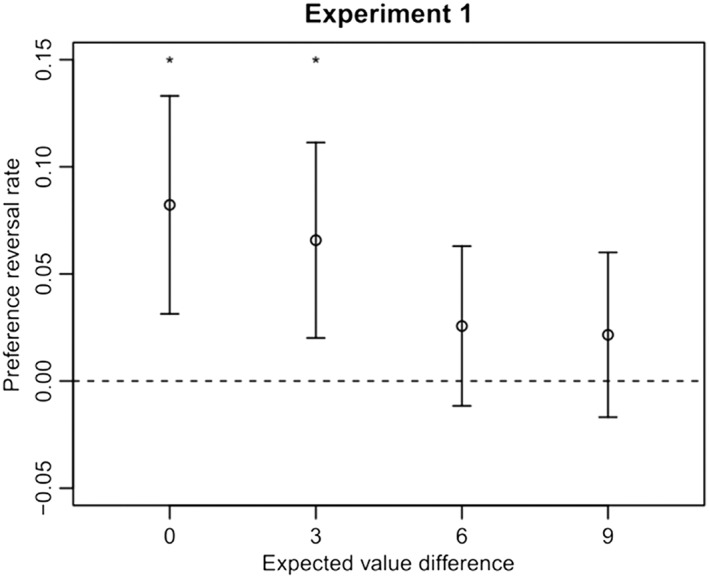
Experiment 1 results. Preference reversal rate for each level of absolute difference in expected value. Error bars are 95% confidence intervals. Asterisks denote that the preference reversal rate was significantly greater than zero

Figure 3 shows that the preference reversal rate decreased as EVD increased. An EVD of zero yielded the highest preference reversal rate and was significantly different from zero assessed with a two‐tailed one‐sample *t* test, *t*(41) = 3.16, *p <* .01, *d* = .50. The preference reversal rate at an EVD of three was also significant, *t*(41) = 2.91, *p* = .01, *d* = .45. 2Contingent on the exclusion of participants with high rates of decoy selections. EVDs of six and nine yielded preference reversal rates not significantly different from zero (*t*(41) = 1.39, *p* = .172, *d* = .21 and *t*(41) = 1.34, *p* = .264, *d* = .17 respectively). A repeated‐measures analysis of variance (ANOVA) revealed a significant effect of EVD on preference reversal rate, *F*(3, 123) = 3.19, *p* = .026, *η* ^2^ = .07.

### Discussion

The results show that, while increasing the difference in expected value between target and competitor prospects reduces the effect of having an asymmetrically dominated decoy in the choice set, the attraction effect is still observed when the EVD is 3 points. In other words, participants can switch to options with lower expected values because of a decoy. The decoy was not able, on average, to persuade participants to switch preference when larger differences in expected value were involved.

## Experiment 2

### Methods

We designed Experiment 2 to be a replication of Experiment 1 using a relative, rather than absolute measure of EVD. We also sought to test the generality of our findings by using a range of different stimuli types. While Experiment 1 shows that participants were sensitive to increases in absolute EVD, it is important to note that an EVD of £3 may be perceived as much larger when going from £3 to £6 than when going from £100 to £103. Therefore, in Experiment 2, we used the percentage increase in expected value from the smaller to the larger prospect as our independent variable. This allowed us to test how robust our findings were and whether they extend to other paradigms that have been used to elicit the attraction effect. See Stimuli section for more details. In Experiment 2, there was no time limit and participants could view all three prospects simultaneously.

#### Participants

One hundred and forty‐three undergraduate psychology students from the University of Manchester volunteered to take part, 50 in Experiment 2a, 52 in Experiment 2b, and 41 in Experiment 2c. Participants received course credits for participating.

#### Design

In each trial, participants chose between a target, competitor, and decoy prospect. Our independent variable was the difference in expected value between the target and competitor prospects. EVD had four levels—0%, 20%, 100%, and 300%—reflecting the percentage increase in expected value from the smaller to larger expected value prospect. Sixteen prospect pairs (Table 2) were created spanning probability and value space, four for each of the independent variable levels.

**Table 2:** Stimuli values used in Experiment 2

Reference prospect	Alternative prospect			
	∆0%	∆20%	∆100%	∆300%
.12 (83)	.24 (42)	.29 (42)	.48 (42)	.96 (42)
.17 (59)	.24 (42)	.29 (42)	.48 (42)	.96 (42)
.59 (17)	.42 (24)	.42 (29)	.42 (48)	.42 (96)
.83 (12)	.42 (24)	.42 (29)	.42 (48)	.42 (96)

Each prospect pair was presented to the participants eight times with one prospect as target, and eight times with the other prospect as target. The decoys had either 20% fewer value points or 20% fewer probability points than the target. The experiment consisted of 256 trials (4 independent variable levels × 4 prospect pairs × 2 decoy positions × 8 repetitions), which were presented in random order. The preference reversal rate (the dependent variable) was calculated in the same way as in Experiment 1. See the Experiment 1 Analysis section for details.

#### Stimuli

Experiment 2a was very similar to Experiment 1, conveying the probabilities and values via the same grid display. The stimuli differed in that the probability grid was not randomised (the green squares appeared as one continuous block).

**Figure 4:**
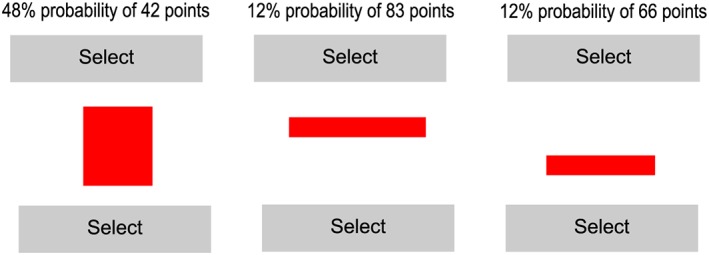
Stimuli used in Experiment 2. The top panel shows the descriptive stimuli from Experiment 2b, while the bottom panel shows the rectangle stimuli in Experiment 2c. [Colour figure can be viewed at wileyonlinelibrary.com]

Experiment 2b presented the stimuli in a manner more similar to the original Wedell (Wedell, 1991) design; sentences were displayed on‐screen to convey the objective probabilities and values of each prospect (top panel of Figure 4). Prospects were presented simultaneously in sentence form ‘*p* probability of *v* points’.

In Experiment 2c, we modelled our stimuli on those used by Trueblood et al. (2013) using a rectangle area instead of expected value (bottom panel of Figure 4). Participants were presented with three rectangles and asked to choose the rectangle with the largest area. The height and width of the rectangles in pixels were the same as probabilities and values used for the prospects in 2a and 2b. As both the area of a rectangle and expected value of our prospects are given by the product of their attributes, it was simple to substitute EVD for area difference.

#### Procedure

For all three experiments, participants were presented with three prospects or rectangles simultaneously on a computer monitor. Below each option, a button marked ‘select’ allowed participants to indicate which prospect they preferred or rectangle they perceived to have the largest area. The on‐screen order of the three stimuli in each trial was randomised; 256 trials were presented in four blocks of 64 trials with an enforced 1‐minute break between blocks.

### Results

#### Experiment 2a

As in Experiment 1, we removed participants whose decoy selection rate exceeded 2.5 times the median absolute deviation (Leys et al., 2013). Seven participants were removed from the following analysis.

**Figure 5:**
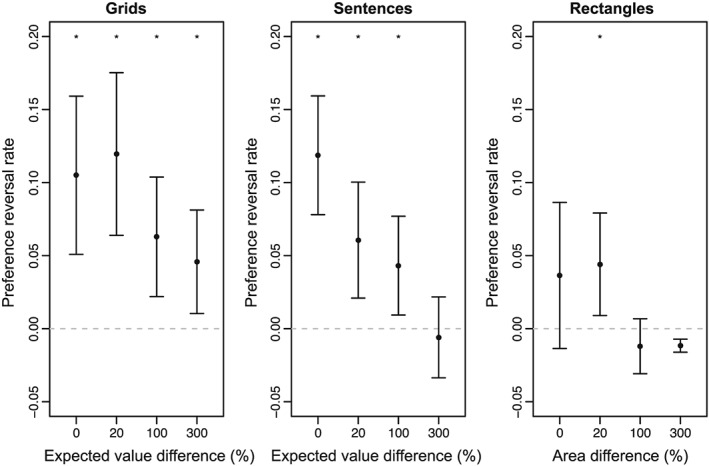
Preference reversal rate for each level of expected value difference in Experiment 2. Error bars are 95% confidence intervals. Asterisks denote reversal rates significantly different from zero

The preference reversal rate was similar in the 0% EVD and 20% EVD conditions but fell in the 100% and 300% EVD conditions. See Figure 5 (left panel). A repeated‐measures ANOVA revealed a significant effect of EVD on preference reversal rate, *F*(3, 126) = 4.99, *p <* .01, *η* ^2^ = .11. Preference reversal rates were significant under a two‐tailed, one‐sample *t* test at zero EVD (*t*(42) = 3.91, *p <* .01, *d* = .60), 20% EVD (*t*(42) = 4.33, *p <* .01, *d* = .66), 100% EVD (*t*(42) = 3.10, *p <* .01, *d* = .47), and 300% EVD (*t*(42) = 2.61, *p* = .01, *d* = .40). 3The significance of the one‐sample *t* tests at 100% and 300% EVD are contingent on the exclusion of participants who had high decoy selection rates.


#### Experiment 2b

Ten participants whose decoy selection rate exceeded 2.5 times the median absolute deviation were removed from the analysis.

The preference reversal rate fell steadily from the 0% EVD condition to close to zero in the 300% EVD condition. See Figure 5 (middle panel). A repeated‐measures ANOVA revealed a significant effect of EVD on preference reversal rate, *F*(3, 123) = 12.24, *p* < .01, *η* ^2^ = .23. Preference reversal rates were significant under a two‐tailed, one‐sample *t* test at zero EVD (*t*(41) = 4.90, *p <* .01, *d* = .91), 20% EVD (*t*(41) = 3.09, *p <* .01, *d* = .48), and 100% EVD (*t*(41) = 2.58, *p* = .01, *d* = .40). At 300% EVD, the preference reversal rate was not significant (*t*(41) = −.434, *p* = .667, *d* = .07). 4The significance of the one‐sample *t* test at 100% EVD is contingent on the exclusion of participants who had high decoy selection rates.


**Figure 6:**
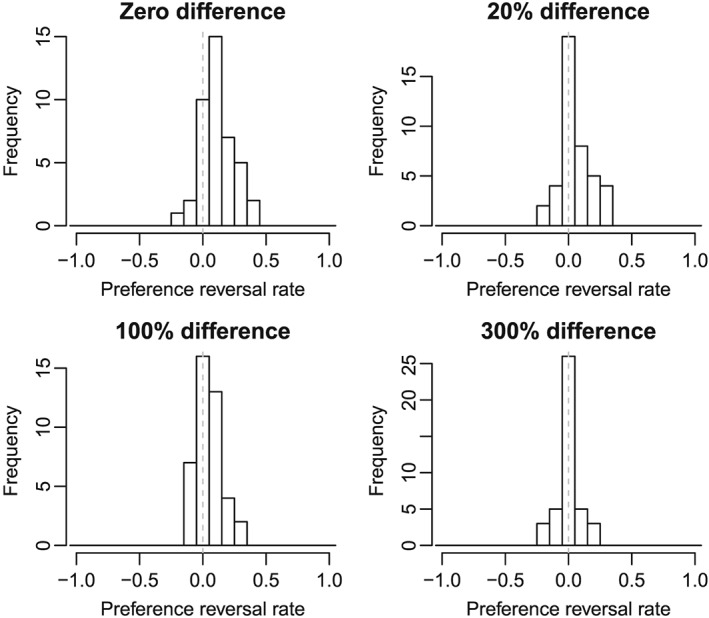
Histograms of preference reversal rate for Experiment 2b. The vertical dashed line at zero represents the expected mean of the distribution if participants were consistent in their choices. Distributions with a positive mean indicate the presence of the attraction effect. See Supporting Information for histograms of Experiments 1, 2a, and 2c

Figure 6 shows the distribution of preference reversal rates. The positive bias in the histograms indicates the presence of the attraction effect, whereas a zero mean would indicate no effect of the decoy. The distributions for the other experiments were similarly shaped (Supporting Information).

#### Experiment 2c

Two participants whose decoy selection rate exceeded 2.5 times the median absolute deviation were removed from the analysis.

The preference reversal rate was low, around 5% in the zero and 20% EVD conditions, falling to around zero in the 100% and 300% area difference conditions. See Figure 5 (right panel). A repeated‐measures ANOVA revealed a significant effect of area difference on preference reversal rate, *F*(3, 114) = 4.91, *p* < .01, *η* ^2^ = .12. The preference reversal rate was not significant under a two‐tailed, one‐sample *t* test at zero area difference (*t*(38) = 1.48, *p* = .148, *d* = .24) but was at 20% area difference (*t*(38) = 2.54, *p* = .02, *d* = .41). Preference reversals were not significant at 100% area difference (*t*(38) = −1.29, *p* = .204, *d* = .21). The preference reversal rate was in the opposite direction to the attraction effect and significant at 300% area difference (*t*(38) = −5.28, *p <* .01, *d* = .84); owing to the very small number of inconsistent choices at this level of area difference, the *t* statistic is disproportionately affected by the few inconsistent and decoy choices. Across all participants, 2496 choices were made in this condition, and 98.6% of those choices correctly identified the rectangle with the larger area. Of the 35 incorrect choices, 31 were decoy choices made when the target alternative was the correct option. We interpret these choices as inattention because the decoy was strictly dominated by the target. Nonetheless, with such a large difference in area between target and competitor, the presence of a decoy alternative does harm the choice share of the target (when the target is the correct alternative).

## General Discussion

The results of both experiments provide the first evidence that the attraction effect can cause people to make choices that do not maximise expected value. While previous experiments have revealed the attraction effect in choice sets that had the same expected value, we have shown that the effect is exhibited when one choice has a higher expected value than the other. Our results also indicate that as the difference in expected value increases, so the attraction effect declines. Given a sufficient difference in expected value, people will not exhibit the attraction effect. In Experiment 1, the preference reversal rate was not significantly different from zero for the two larger differences in expected value. Likewise in Experiment 2, preference reversal rates fell as the difference in expected value increased.

Differences in the rate of preference reversal between Experiments 1 and 2a, which used similar stimuli, are likely due to the lack of time pressure in Experiment 2. Time pressure in attraction effect experiments has been shown to reduce the size of the effect (Pettibone, 2012). It should be noted that we use EVDs as a proxy for expected utility differences, and a useful replication would be to test these results using a direct expected utility manipulation. It is also apparent that the results of Experiment 2c reveal a smaller preference reversal rate than the other paradigms. The size of the change is however consistent with that reported in previous work using area judgments (Trueblood et al., 2013). Our results from Experiment 2c, together with those from Trueblood et al. (2013), do suggest that the attraction effect can impact upon perceptual judgments. These data are in contrast to those reported by Frederick et al. (2014) who failed to find an effect in a rectangle area judgment task. However, more recent studies have replicated the effect using similar stimuli in rhesus macaques (Parrish, Evans, & Beran, 2015) and in children (Zhen & Yu, 2016). Frederick et al. also argue that the attraction effect may not be elicited in gambles when these are represented perceptually rather than explicitly. We have found an attraction effect in gambles represented both explicitly and perceptually. Using very similar designs, in both representations, we found that differences in expected value decrease the attraction effect, and also that the effect can persist when differences are present. Overall, our data suggest that the attraction effect is in fact a robust phenomenon.

This finding poses a more severe problem for rational theories of choice than previous preference reversal studies, as it shows not just the violation of an axiom but also the potential for a lower value outcome as a result of the behavior. This second point should be much more important for rational theorists than the axiomatic violation. That the attraction effect occurs in the face of EVDs addresses concerns that have been expressed over a number of years in the literature (Anderson, 1990; Hahn & Harris, 2014; Roth, 1996). Roth, as quoted in the Introduction to the current article, is concerned less with the existence of preference reversals and more with quantifying how important they might be, with importance being a function of their robustness in the presence of differences in expected value. Similarly, Hahn and Harris (2014) have critiqued logical demonstrations of irrationality in human decision making for often failing to examine the cost of these phenomena. This emphasis on the cost of such behaviors is theoretically important because proponents of rational accounts are more concerned with the quality of the outcome than the process that produces the decision. Anderson makes the following point with respect to violations of transitivity in his introduction to rational analysis:

A question that is rarely asked is whether there is really a cost associated with the purported irrationality. If a person prefers A to B, B to C and C to A, but there are no differences among A, B, and C in their adaptive value, then the intransitivity does not violate the adaptive principle of rationality (Anderson, 1990, p. 32).

Our results suggest that the attraction effect might properly be considered in violation of value‐maximising models because it can lead people to choose, within some tolerance, the lower expected value of two alternatives. One interpretation might be that this reveals how our bounded decision processes prevent us from achieving normative standards in decision making. Certainly, data showing ordinal comparisons, as observed by Noguchi and Stewart (2014), suggest that people are engaged in a process other than expected value calculation (although these data do not yet tell us what happens across the full range of EVDs). Existing accounts of the attraction effect often state that it is problematic for value‐maximising theories because it violates the key axioms of regularity and independence from irrelevant alternatives. In these accounts, the behavior is interpreted as a side effect of larger decision‐making systems or heuristics, and not as a behavior that is beneficial.

In contrast, an alternative explanation is that preference reversals are rational (Bordley, 1992; Howes et al., 2016; McNamara, Trimmer, & Houston, 2014; Shenoy, & Yu, 2013; Trimmer, 2013). Rather than taking axiomatic violations as evidence that people's cognitive bounds prevent them from achieving normatively correct decisions, we can assume the cognitive bounds and ask what the optimal decision should be given those limitations (Howes, Lewis, & Vera, 2009; Lewis, Howes, & Singh, 2014). In another recent work, we apply this approach to show that assuming the constraint of noisy expected value calculation is sufficient to make the attraction effect beneficial (Howes et al., 2016). This is because the presence of the decoy provides information about the likely values of the other alternatives where all the alternatives are regarded as samples from a common environment. Consider the situation where three prospects are available but nothing is known about their attribute values. In this scenario, each option is equally likely to have the highest expected value. However, if we know the ordinal relations between the attribute values (but not their cardinal values), we can determine which option is most likely to have the highest expected value. In the case of the attraction effect, the ordinal relations that define the alternatives are *p*(*T*) *> p*(*D*) *> p*(*C*) and *v*(*C*) *> v*(*T*) *> v*(*D*), where *p*(*X*) indicates the probability of success for lottery *X* and *v*(*X*) indicates the value of a win for lottery *X*. Given this information, there are three possible expected value relations in which *T* dominates *D* on both attributes: *EV*(*T*) *> EV*(*D*) *> EV*(*C*), *EV*(*T*) *> EV*(*C*) *> EV*(*D*), and *EV*(*C*) *> EV*(*T*) *> EV*(*D*). In two of these, the target has higher expected value than the competitor. There is therefore a two‐thirds probability that the target is a better choice than the competitor given just the ordinal information. The only requirement for this information to be useful is that the decision maker does not have a noise‐free estimate of expected value—a requirement that seems incontrovertible. Using only this assumption of noisy perception, Howes et al. (2016) show that it is rational to update the expected value estimate with the information provided by the ordinal relations. This process naturally produces the attraction effect, as well as other contextual preference reversals including the similarity and compromise effects. It should also be noted that this explanation is consistent with the attraction effect declining as EVD increases. This is because a large difference in expected value will resolve the ambiguity caused by noisy estimation and reduce the impact of considering the ordinal relations. More broadly, in a particular choice instance, a person may appear locally irrational by virtue of choice axiom violations, but when the global context and relevant priors or cognitive constraints are taken into account, the behavior may be rational.

By examining the attraction effect in an environment in which it is possible to *not* value‐maximise, we have established a relationship between the effect and value maximisation (as recommended by Anderson, 1990; Hahn & Harris, 2014; Roth, 1996). Rather than take phenomena such as the attraction effect as evidence that rational value‐maximising accounts do not explain human decision making, a more fruitful approach would be to consider *why* a behavior occurs as well as *how* it occurs. Attempting to answer the why question is the objective in value‐maximising accounts. This type of explanation also has the benefit of providing a constraint on the number of plausible processes (as only a small subset may be optimal) that can produce a behavior. It is important therefore that apparently irrational phenomena are not seen as validating process accounts over rational accounts. Instead, these phenomena might be better understood by allowing process and rational accounts to complement one another in furthering our understanding of decision making.

## Supporting information

Figure S1: Histograms of the preference reversal rate for Experiment 1. The vertical dashed line at zero represents the expected mean of the distribution if participants were consistent in their choices. Distributions with a positive mean indicate the presence of the attraction effect
Figure S2: Histograms of the preference reversal rate for Experiment 2a. The vertical dashed line at zero represents the expected mean of the distribution if participants were consistent in their choices. Distributions with a positive mean indicate the presence of the attraction effect
Figure S3: Histograms of the preference reversal rate for Experiment 2c. The vertical dashed line at zero represents the expected mean of the distribution if participants were consistent in their choices. Distributions with a positive mean indicate the presence of the attraction effect

Supporting info item
