# Supplementary material for: The Effect of Expected Value on Attraction Effect Preference Reversals
Source: J Behav Decis Mak. 2016 Dec 19;30(4):785–93. doi: 10.1002/bdm.2001 (PMC5637901; doi:10.1002/bdm.2001)
Supplement: Supplementary file 1 — Supporting info item [file BDM-30-785-s001.pdf]

The Effect of Expected Value on Attraction Effect Preference Reversals - Supporting Information

George D. Farmer, Paul A. Warren, Wael El-Deredy and Andrew Howes

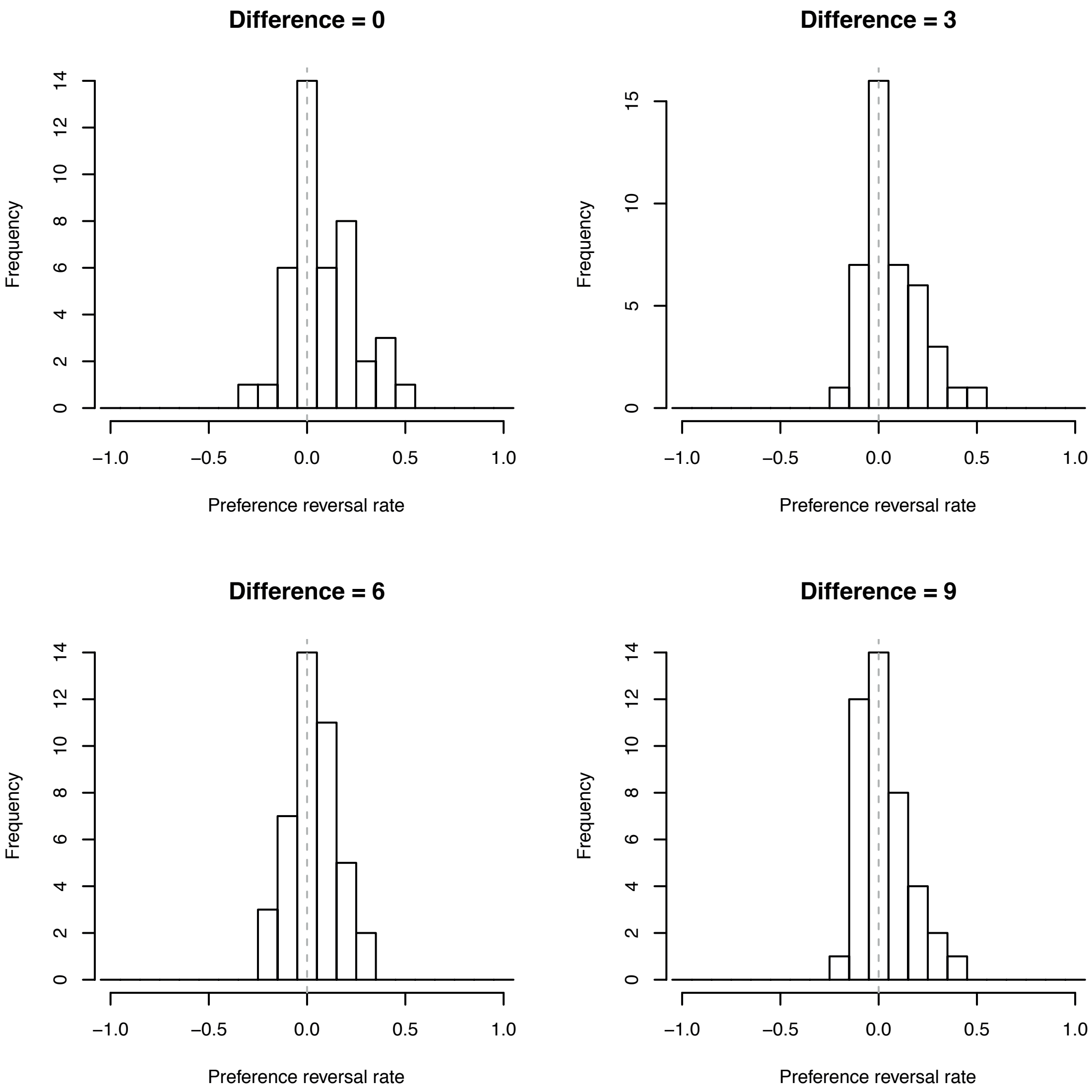

Figure S1: Histograms of the preference reversal rate for Experiment 1. The vertical dashed line at zero represents the expected mean of the distribution if participants were consistent in their choices. Distributions with a positive mean indicate the presence of the attraction effect

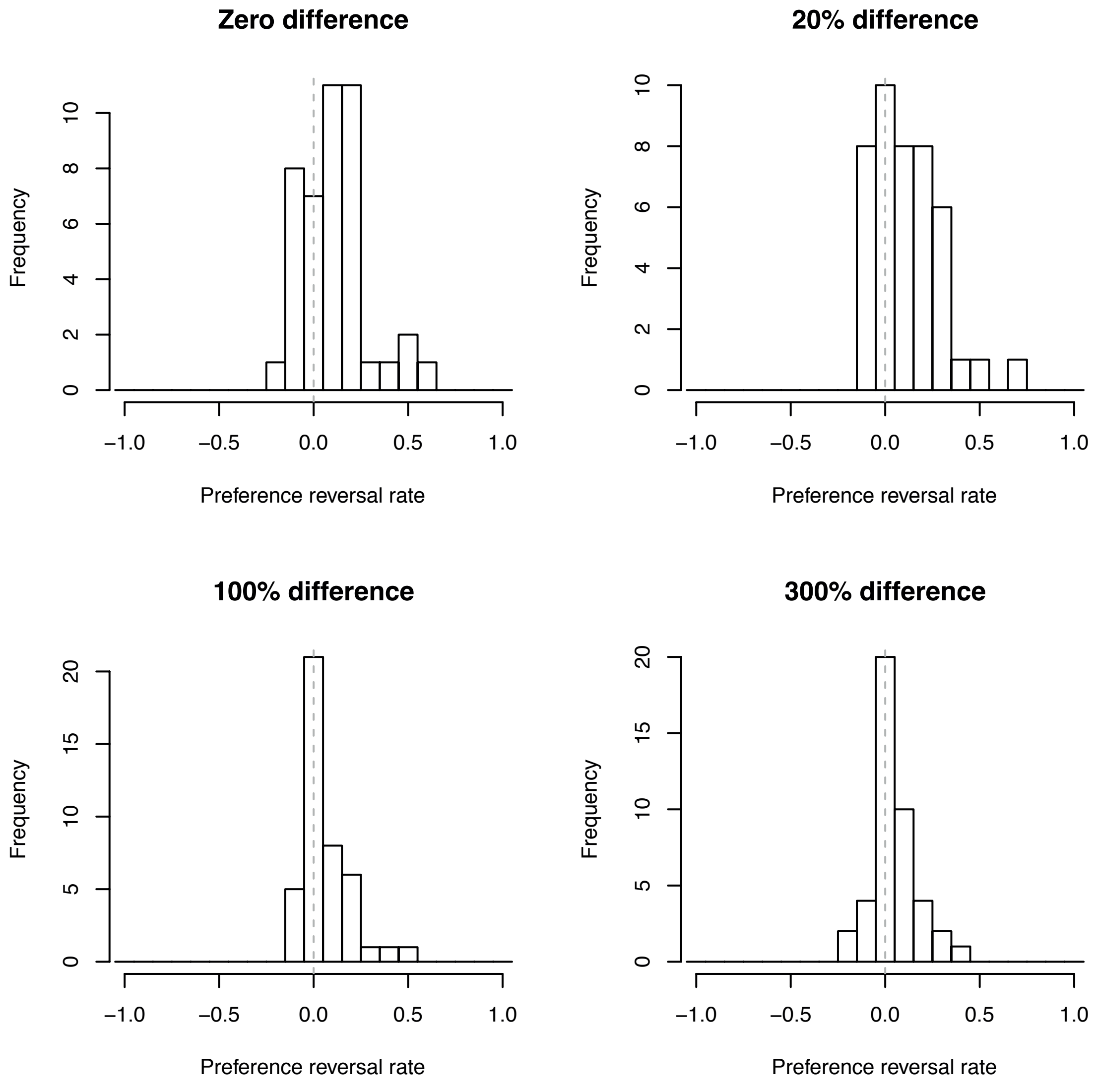

Figure S2: Histograms of the preference reversal rate for Experiment 2a. The vertical dashed line at zero represents the expected mean of the distribution if participants were consistent in their choices. Distributions with a positive mean indicate the presence of the attraction effect

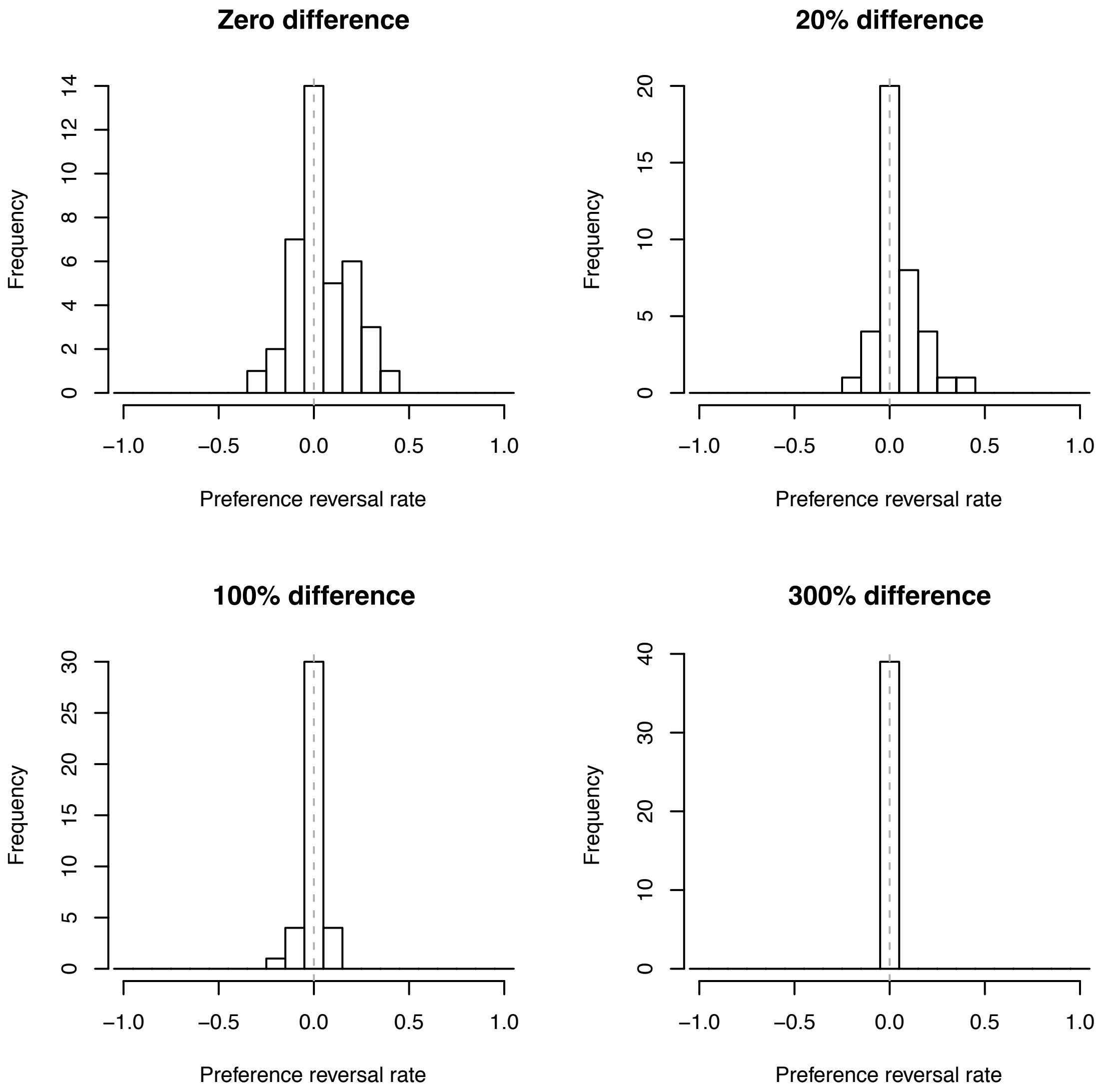

Figure S3: Histograms of the preference reversal rate for Experiment 2c. The vertical dashed line at zero represents the expected mean of the distribution if participants were consistent in their choices. Distributions with a positive mean indicate the presence of the attraction effect
